# *In Vitro* Anthelmintic Activity of Saponins from *Medicago* spp. Against Sheep Gastrointestinal Nematodes

**DOI:** 10.3390/molecules25020242

**Published:** 2020-01-07

**Authors:** Michela Maestrini, Aldo Tava, Simone Mancini, Doriana Tedesco, Stefania Perrucci

**Affiliations:** 1 Department of Veterinary Sciences, University of Pisa, viale delle Piagge 2, 56124 Pisa, Italy; michela.maestrini@phd.unipi.it (M.M.); simone.mancini@unipi.it (S.M.); 2 CREA Research Centre for Animal Production and Aquaculture; viale Piacenza 29, 26900 Lodi, Italy; aldo.tava@crea.gov.it; 3 Department of Environmental Science and Policy, University of Milano, via Celoria 2, 20133 Milano, Italy; doriana.tedesco@unimi.it

**Keywords:** saponins, prosapogenins, *Medicago* spp., sheep, gastrointestinal strongyles, ‘in vitro’ anthelmintic activity

## Abstract

Gastrointestinal strongyle nematodes (GIS) are included among the most important parasites of small ruminants. The widespread drug resistance and drug residues in products of animal origin have increased the interest in the search for natural compounds with anthelmintic activity as a valid alternative to current synthetic drugs. The aim of the present investigation was to test the ‘in vitro’ anthelmintic activity of saponins and prosapogenins from different *Medicago* species, selected for their importance as a forage crop worldwide for animal feeding. From these plants, saponin mixtures were extracted, purified and used at scalar concentrations to evaluate their anthelmintic activities against sheep gastrointestinal strongyles (GISs), by the egg hatch test. Treated and untreated controls were used as the comparison. Data were statistically analyzed, and EC_50_ and EC_90_ were also calculated. All saponins and prosapogenins showed inhibiting effects on GIS eggs in a concentration-dependent manner. At higher concentrations, most of them showed an efficacy comparable to the reference drug (Thiabendazole 3 µg/mL) (*P* < 0.001). With 1.72 mg/mL EC_50_ and 3.84 mg/mL EC_90_, saponin from *M. polymorpha* cultivars Anglona was the most active. Obtained results encourage further studies aimed at evaluating the efficacy ‘in vivo’ of saponins which resulted as most effective ‘in vitro’ in this study.

## 1. Introduction

Gastrointestinal strongyles (GIS) are considered one of the most common causes of economic losses and disease in small ruminant breeding [1]. These parasites are nematodes belonging to the Strongylida order, and localize in the gastrointestinal tract of small ruminants. Infected animals may show reduced growth, cachexia, weakness, anemia and diarrhea, that may lead to poor reproductive and productive performances and death [2]. In the last decades gastrointestinal strongyles infections have been primarily controlled with synthetic drugs belonging to different chemical classes, i.e., benzimidazoles, imidazothiazole/tetrahydroxypyrimidines, macrocyclic lactones, amino-acetonitrile derivates and spiroindoles [1]. However, the regular and sometimes excessive use of anthelmintics has contributed to the onset of drug resistance, which is now widespread worldwide [3], limiting the effectiveness of synthetic drugs for the control of gastrointestinal nematode infections [4,5]. Confirming this, there are reports of the reduced efficacy of some recently commercialized anthelmintics, such as monepantel or derquantel against the GIS species *Haemonchus contortus* [6]. Therefore, to date, many anthelmintics prove to be inefficacious, as well as polluting [7]. Another important issue linked to the use of synthetic drugs is that their residues can be found in products of animal origin, such as meat and milk [8].

For the control of GIS there are various environmentally sustainable, non chemical approaches that can limit the use of synthetic drugs in ruminants, such as vaccination, biological control, nutritional supplementation and grazing management, including pasture rotation [9], but in most cases the complementary aid of an anthelminthic treatment is still required [10]. For this reason, there is an increasing interest in natural compounds with anthelmintic activity, such as plant extracts and plant-derived compounds, with the aim to find a valid alternative to current synthetic drugs, or that can be used as a model for the synthesis of new drugs. Several previous ‘in vitro’ and ‘in vivo’ studies in small ruminants have shown that different plant extracts and pure compounds of plant origin possess anthelmintic properties against GIS species, including two of the most pathogenic common nematodes of small ruminants, i.e., *Haemonchus contortus* [11,12] and *Teladorsagia circumcincta* [13]. The anthelmintic activity of these plant extracts is related to the presence of biologically active metabolites such as condensed tannins, flavonoids, steroids, terpenoids, alkaloids and saponins [14,15]. Among these, saponins are important secondary metabolites from plants, and are considered as potential anthelmintic natural compounds [16,17]. It is reported that fractions rich in steroidal and triterpenic saponins from *Agave sisalana* have an ‘in vitro’ ovicidal effect against the nematodes of goats [18]. More recently, the ‘in vitro’ nematicidal potential of saponins from different *Medicago* spp. against donkey nematodes was demonstrated [19]. Biological effects of saponins are normally ascribed to their specific interaction with cell membranes [20], causing changes in cell permeability. By affecting some cell membrane components, saponins induce the formation of micelle-like aggregates that disrupt membrane functionality and cause lysis [21]. For nematodes, saponins have been associated with the formation of complexes with cellular membrane components present in different stages of the nematode life cycle, leading to an increase in membrane permeability and causing these parasites to die [21,22]. Saponins are detected in many plant species, including the genus *Medicago*, in which they are triterpenic pentacyclic glycosides with a wide range of biological properties, including antimicrobial, fungicidal, nematicidal, cytotoxic and insecticidal activities [20,23,24,25,26].

The aim of the present investigation was to test ‘in vitro’ the anthelmintic activity of saponins and prosapogenins from different *Medicago* species, selected for their importance as a forage crop worldwide for animal feeding. Alfalfa, *M. sativa* L. and burr medic, *M. polymorpha* L. have been considered as species that have an agronomic relevance in Mediterranean environments [27]. From these forage plants, saponin mixtures were extracted, purified and used at different concentrations to assess their ‘in vitro’ anthelmintic activities against GIS of sheep, by using the egg hatch test (EHT), that evaluates the ability of a compound to inhibit the development and the hatch of GIS eggs.

## 2. Results

### 2.1. Saponin Composition

**Figure 1:**
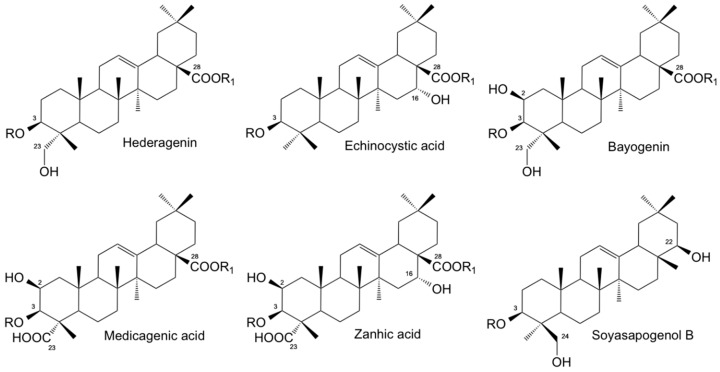
Chemical structure of the most abundant sapogenins (R = R_1_ = H) detected in the *Medicago* spp. used in this study. R = R_1_ = sugar or sugar chain: bidesmosidic saponins; R = sugar or sugar chain, R_1_ = H: monodesmosidic saponins.

The compositional profile of *Medicago* saponin extracts used in this study differed according to the plant species [28,29,30]. Crude saponins were obtained from the *Medicago* species under investigation as whitish powder in a purity of about 90% and in a different yield: *M. polymorpha* cv. Anglona 2.1% dry matter (DM), *M. polymorpha* cv. Santiago 1.7% DM and *M. sativa* cv. Equipe 1.5% DM. Figure 1 shows the chemical structure of the most abundant saponins/sapogenins detected in the different *Medicago* extracts. The content of the most abundant sapogenins, obtained after acid hydrolysis of the corresponding glycosides, is reported in Table 1. 

Saponins from *M. sativa* were characterized by a higher amount of medicagenic and zanhic acids (Figure 1), quoted as 47.2% and 25.5% of the total sapogenins, respectively. Hederagenin (Figure 1) was instead the dominant sapogenin in *M. polymorpha* cv. Santiago, representing 88.3% of the total aglycones, while echinocystic acid (90.1%) (Figure 1) was the dominant sapogenin detected in *M. polymorpha* cv. Anglona. Soyasapogenol B, the aglycone moiety of soyasaponin I, a saponin commonly present in Leguminosae, was detected in all samples, although in a different amount. From the high-performance liquid chromatography (HPLC) analyses of saponins (data not shown), and by comparison with authentic reference compounds previously identified in the *Medicago* spp. [30,31,32], all the saponin mixtures here evaluated were mainly constituted by bidesmosidic type saponins (70%–80%). *M. sativa* prosapogenins, obtained after basic hydrolysis of the corresponding saponins, were instead entirely made up by monodesmosides and possess the same sapogenin composition as *M. sativa* from which they were obtained. 

### 2.2. ‘In Vitro’ Anthelmintic Activity of Saponin Mixtures

**Figure 2:**
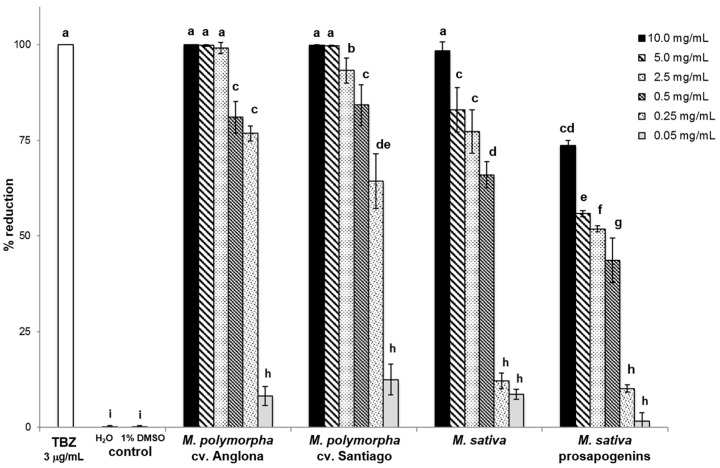
Percentages of egg hatch reduction found for the saponin and prosapogenin mixtures tested at different concentrations (from 10 to 0.05 mg/mL) compared to positive (thiabendazole (TBZ) 3 µg/mL) and negative (H_2_O and H_2_O-1% dimethyl sulfoxide (DMSO)) controls. a–i represent a statistical differences.

Results of the ‘in vitro’ anthelmintic activity of the saponin mixtures tested at different concentrations against sheep GIS eggs are reported in Figure 2. All the saponin mixtures used in this study showed inhibiting effects against GIS eggs in a concentration-dependent manner. More specifically, 10, 5 and 2.5 mg/mL *M. polymorpha* cv. Anglona, 10 and 5 mg/mL *M. polymorpha* cv. Santiago and 10 mg/mL *M. sativa* saponins were able to inhibit almost 100% of GIS eggs. For these saponin samples the same anthelmintic effects were recorded as for the reference drug, thiabendazole (TBZ), tested at 3 µg/mL (*P* < 0.001). 

A very good inhibition activity (93%) was also observed for *M. polymorpha* cv Santiago at 2.5 mg/mL, while the efficacy of 0.5 and 0.25 mg/mL *M. polymorpha* cv. Anglona, 0.5 mg/mL *M. polymorpha* cv Santiago, 5 and 2.5 mg/mL *M. sativa* saponins and 10 mg/mL *M. sativa* prosapogenins ranged between 73% and 84% (Figure 2). Egg hatching inhibition values comprised between 43% and 66% were observed for *M. polymorpha* cv. Santiago at 0.25 mg/mL, *M. sativa* saponins at 0.5 mg/mL and *M. sativa* prosapogenins in the range 5–0.5 mg/mL concentration. All other saponin solutions (0.05 mg/mL *M. polymorpha* cv. Anglona and cv. Santiago, 0.25 and 0.05 mg/mL *M. sativa* saponins and 0.25 and 0.05 mg/mL *M. sativa* prosapogenins) showed a very low efficacy (2%–12.5%), but statistically different (*P* < 0.001) from the untreated controls for which an egg hatch inhibition less than 1% was registered (Figure 2). 

From fecal cultures, *Trichostrongylus* spp. (40%), *Oesophagostomum* spp. (20%), *Cooperia* spp. (20%), *Haemonchus* spp. (10%) and *Chabertia* spp. (10%) GIS genera were identified. Negative effects of tested saponins against the different nematode genera were approximately similar.

For the calculation of the EC_50_ and EC_90_, the best regression line was performed with a logarithmic curve (natural logarithm), with an R^2^ ranging from 0.87 to 0.93. With 1.72 mg/mL EC_50_ and 3.84 mg/mL EC_90_, *M. polymorpha* cv. Anglona was the most active saponin, followed by *M. polymorpha* cv Santiago (1.77 mg/mL EC_50_ and 4.00 mg/mL EC_90_) and *M. sativa* saponins (2.34 mg/mL EC_50_ and 4.71 mg/mL EC_90_). Showing 3.33 mg/mL EC_50_ and 8.13 mg/mL EC_90_, *M. sativa* prosapogenins were the less effective compounds.

## 3. Discussion

Results obtained in this study show that all *Medicago* saponins and prosapogenins evaluated in this study have ‘in vitro’ inhibiting effects against sheep GIS eggs, although with a different level of efficacy, with *M. polymorpha* saponins as the most active. A dose dependent inhibition effects on egg hatch and development was also observed (Figure 2). 

Moreover, the EC_90_ and EC_50_ values found for the most active saponins in this study are similar to those reported for other active plant compounds [33]. These results are in agreement with previously reported data, confirming a high ovicidal activity of saponins from *Medicago* spp. [19]. As observed by [19], when tested at the 10 mg/mL concentration against donkey GIS eggs, saponins from *Medicago* spp. show a high activity (80%–100% egg hatch reduction) with *M. polymorpha* cv. Anglona and *M. sativa* as the most active. Differences in saponin activity between sheep and donkey GIS eggs can probably be ascribed to a different susceptibility of sheep and donkey GIS species to tested saponins. The available data from literature also confirm the ‘in vitro’ anthelmintic activity of this class of compounds against ruminant GIS. The saponins aescin and digitonin [17] and saponin fractions contained in *Zizyphus joazeiro* [16], *Phytolacca icosandra* [34] and *Agave sisalana* [18] showed ‘in vitro’ ovicidal activity against the nematodes of small ruminants. Similarly, the ‘in vitro’ ovicidal action of *Combretum molle* against eggs of *H. contortus* from sheep and of *Ipomoea chiliantha*, *Tocoiena formosa* and *Aspilia latissima* against the eggs of *Haemonchus placei* from cattle, have been attributed to their saponin content [33,35]. Biological effects of saponins are normally ascribed to their specific interaction with the cell membrane, causing changes in the cell permeability [20,36,37]. By this way, saponins may penetrate inside the GIS eggs, altering some biological functions and preventing the normal development of eggs, thus leading to the inhibition of the development of eggs definitively, as proposed by [16]. It has been also hypothesized that these compounds may be able to interfere with enzymatic pathways involved in larval development, which results in larval death [16].

Data obtained in this study on the structure–activity relationship showed that all the *Medicago* saponins are active compounds against GIS eggs, independently of the involved genins. Comparing results between saponins and related prosapogenins, data here presented indicated that *M. sativa* prosapogenins (monodesmosides) resulted less active than the related saponins (bidesmosides). These results agree with previously reported data on GIS eggs from donkey [19]. 

The ‘in vitro’ efficacy of saponins from *Medicago* spp. in inhibiting the hatching of GIS eggs of sheep and donkey [19], encourage further studies aimed at evaluating their efficacy ‘in vivo’ as new anthelmintic compounds, as nutraceuticals or as a means to inhibit the environmental development of GIS eggs in order to lower pasture contamination. However, various aspects related to their potential toxicity should be considered. Saponins, in fact, as well as tannins, if ingested in large quantities, are potentially toxic, and may diminish the digestibility of feed [38,39]. However, if taken in moderate concentrations, they can improve nutritional effects [40], and at the same time can reduce the parasitic burden [34]. A previous study [41] showed that a diet containing 1.5% of saponins from *Quillaja saponaria* bark may reduce by 38.8% the sheep fecal egg count (FEC) of GIS eggs when compared to untreated animals. The high ‘in vitro’ activity of the *Medicago* saponins evaluated in the present study against sheep GIS eggs may suggest their potential ‘in vivo’ efficacy at non-toxic dosages.

## 4. Materials and Methods

### 4.1. Plant Material, Extraction, Purification and Characterization of Saponin Mixtures

*Medicago* plants used in this study were grown at the Research Center for Animal Production and Aquaculture (CREA-ZA, Lodi, Italy). Aerial parts from *M. polymorpha* cv. Santiago, *M. polymorpha* cv. Anglona, and *M. sativa* cv. Equipe were utilized for saponin processing. Leaves were separated from stems and dried at 40 °C to a constant weight. All samples were ground and used for the successive extractions. Saponins were extracted and purified following general procedures, as previously reported [30,31,32]. Powdered plant materials (150 g) were separately defatted with CHCl_3_ in a Soxhlet apparatus for 24 h. Defatted material (100 g) was separately extracted with 80% MeOH under reflux for 24 h. The solvent was removed under reduced pressure, and the residue was re-suspended in 30% MeOH. The solution was applied onto a 100 × 60 mm RP-18 (40–63 μm) column, preconditioned with 30% MeOH. Elution was carried out with 30% MeOH (500 mL) to remove sugars and some phenolics. 

Total saponins were then eluted with 90% MeOH (400 mL) and dried under vacuum. 2.06 g of saponins were obtained from *M. polymorpha* cv. Anglona (2.1% yield), 1.72 g of saponins were obtained from *M. polymorpha* cv. Santiago (1.7% yield), while 1.48 g of saponins were obtained from *M. sativa* cv. Equipe (1.5% yield). In addition, saponins from *M. sativa* were subjected to basic hydrolysis [23] to obtain the related prosapogenins, which were also evaluated in this study. All samples were dissolved in H_2_O-5% dimethyl sulfoxide (DMSO), solutions were properly diluted with H_2_O and used in the bioassay at different final concentrations from 0.05 to 10.0 mg/mL. The saponin mixtures, obtained as whitish powders of about 90% purity, were analyzed by thin layer chromatography (TLC), as previously described [30]. In addition, extracted and purified saponins were characterized for their qualitative and quantitative aglycone composition by gas chromatography (GC) and gas chromatography/mass spectrometry (GC/MS) analyses of derivative sapogenins obtained after acid hydrolysis, as already reported [42]. To obtain information on saponin composition and purity, the saponin mixtures were then analyzed by high-performance liquid chromatography (HPLC), using an external standard method [30], and the results compared with standards of previously purified and identified saponins and data from literature [30,31,32].

### 4.2. Nematode Egg Collection, Purification and Suspension

Purified GIS eggs were obtained from fecal samples of naturally infected sheep from an organic farm in Tuscany (central Italy). For transport to the laboratory, fecal samples were placed in sealed and refrigerated bags. Fecal microscopic analysis was performed using the Mini-FLOTAC technique [43], with a sensitivity of 20 eggs per gram of feces (EPG). Recovery and suspension of GIS eggs were performed within 3 h of collection using previously reported methods [44] with some modifications. More specifically, 30 g of feces were mixed with distilled water and then centrifuged in 50 mL tubes at 900× *g* for 5 min. The supernatant was than eliminated and an NaCl saturated solution was added to the sediment and centrifuged at 170× *g* for 5 min. The supernatant was collected and centrifuged for the last time with distilled water in 15 mL tubes at 900× *g* for 5 min. The supernatant was eliminated in order to obtain the sediment containing the purified eggs that were inspected microscopically to exclude any embryonation that had begun, then they were counted, diluted in distilled water to the final concentration of about 400 eggs/mL and used immediately in the bioassay. Finally, fecal cultures were also performed by using the same pooled fecal samples employed for obtaining gastrointestinal strongyles eggs, to the aim of identifying GIS nematodes at the genus level. Fecal samples were cultured in an incubator at 25 °C for seven days, and larvae were recovered by the Baermann technique and identified according to the key and description given by [45].

### 4.3. Evaluation of the ‘In Vitro’ Anthelmintic Activity of Saponins

The Egg Hatch Test (EHT) was performed to evaluate the ‘in vitro’ anthelmintic activity of saponin and prosapogenin samples. In EHT the effectiveness of the substances is evaluated based on their ability to inhibit the development and hatching of parasite eggs [44,46]. In the present work the EHT was carried as in the previously reported methods [35,44,46], with some minor modifications. In detail, 24-well, flat-bottomed microplates were used, and 0.5 mL of distilled water containing approximately 200 eggs were placed in each well. All the test samples were treated with 0.5 mL of the different saponin solutions to obtain the reported final saponin concentration. Positive controls contained the same amount of GIS eggs and the anthelmintic drug TBZ (2-(4-Thiazoly) Benzimidazole (Thiabendazole) minimum 99%, Sigma S.r.l., Milan, Italy) at the final concentration of 3 µg/mL in 1% DMSO. Two different negative controls were prepared by adding to the egg suspension 0.5 mL of distilled water and 0.5 mL of 1% DMSO. The plates were then incubated at 25 °C in darkness and 80% humidity for 48 h, and the number of eggs and of the first-stage larvae (L1) in each well was microscopically counted. The percentage of egg hatch reduction was calculated using the following formula:

number of eggs/(number of L1 + number of eggs) × 100(1)

All experiments were performed in triplicate in three independent assays.

### 4.4. Statistical Analysis

Results from all experiments were statistically analyzed and compared. Statistical analysis was performed using the Statistical Analysis System (SAS) program. To perform the statistical analysis of the obtained data, a one-way analysis of variance (ANOVA) test with 5% significance (*P* < 0.05), was used. Significant results were further tested with the Tukey post-hoc test (*P* < 0.05). The determination of EC_50_ and EC_90_ concentrations was done through non-linear regression analysis by using XLSTAT^®^.

### 4.5. Ethical Declaration

This study has not included animal experiment. Sheep fecal samples used for the evaluations performed in this study have been collected with the consensus of the farm owner. Authors declare that the work has been carried out in adherence to a high standard of veterinary care.

## Figures and Tables

**Table 1:** Percentage composition of the most abundant sapogenins detected in the *Medicago* saponin mixtures used in this investigation. For chemical structures see Figure 1.

Sapogenin	*M. polymorpha* cv. Anglona	*M. polymorpha* cv. Santiago	*M. sativa* cv. Equipe
Hederagenin	3.6	88.3	1.1
Echinoystic acid	90.1	2.8	−
Bayogenin	0.2	3.2	1.8
Medicagenic acid	−	−	47.2
Zanhic acid	−	−	25.5
Soyasapogenol B	2.1	3.6	13.3
